# Prognosis of unresected versus resected early‐stage pulmonary carcinoid tumors ≤3 cm in size: A population‐based study

**DOI:** 10.1002/cam4.7311

**Published:** 2024-06-10

**Authors:** Xiongfei Li, Fanfan Fan, Xuewang Jia, Lingqi Yang, Jinling He, Quanying Tang, Weibo Cao, Ji Che, Song Xu

**Affiliations:** ^1^ Department of Lung Cancer Surgery, Lung Cancer Institute Tianjin Medical University General Hospital Tianjin China; ^2^ Tianjin Key Laboratory of Lung Cancer Metastasis and Tumor Microenvironment, Lung Cancer Institute Tianjin Medical University General Hospital Tianjin China; ^3^ Department of Thoracic Surgery and State Key Laboratory of Genetic Engineering Fudan University Shanghai Cancer Center Shanghai China; ^4^ Department of Oncology, Shanghai Medical College Fudan University Shanghai China

**Keywords:** observation, pulmonary carcinoid, SEER, surgery

## Abstract

**Purpose:**

The observation‐based prognosis, rather than resection, for small carcinoid tumors is still unclear. This lack of clarity has important implications for counseling elderly patients or patients for whom surgical resection poses a high risk. This study compared the outcomes of observation and surgical resection in patients with pulmonary carcinoid (PC) tumors ≤3 cm in size without metastasis.

**Methods:**

Data of patients with PC tumors with ≤3 cm in diameter and without lymph node and distant metastases were retrieved from Surveillance, Epidemiology, and End Results (SEER) registry. To reduce the inherent bias of retrospective studies, propensity score matching analysis was performed. Overall survival (OS) and lung carcinoid‐specific survival (LCSS) were analyzed using Kaplan–Meier plots. Multivariate analysis was used to determine predictors of LCSS in different size subgroups.

**Results:**

In total, 4552 patients with early‐stage PCs ≤3 cm in diameter, including 435 (9.56%) who were observed and 4117 (90.44%) treated by surgery, were recruited. Patients with surgery had significantly better OS and LCSS than those who were observed. However, patients with observation had comparable LCSS to those with surgery for PCs with tumor diameters ≤1 cm. Multivariate analysis indicated that surgical resection was an independent prognostic factor for LCSS in 1 cm < tumors ≤2 cm, and 2 cm < tumors ≤3 cm groups, but not for tumors ≤1 cm in diameter.

**Conclusion:**

Surgical resection of small PCs is associated with a survival advantage over observation. However, for early PCs ≤1 cm in diameter, observation may be considered in patients with high risk for surgical resection.

## Graphical abstract

This is the first cohort study with the largest number of early‐stage pulmonary carcinoids (PCs) to focus on the comparison of outcomes between observation and surgical resection in patients with early‐stage PC tumors ≤3 cm in size without lymph node or distant metastasis. After propensity score matching, patients in the observation group had comparable lung carcinoid‐specific survival to those in the surgery group for PCs with tumor diameters ≤1 cm, which indicated that observation may be considered in patients with high risk for surgical resection for early PCs ≤1 cm in diameter.

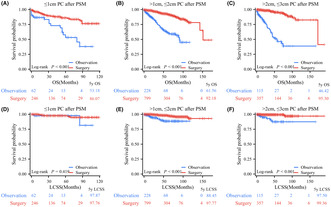

## 1 INTRODUCTION

Pulmonary carcinoids (PCs), originating from bronchial epithelial Kulchitsky cells with neuroendocrine activity, are rare tumors that account for approximately 1%–2% of all lung neoplasms.^1^, ^2^, ^3^, ^4^ PCs are a type of lung neuroendocrine tumor and histopathologically classified as typical carcinoids (TCs) and atypical carcinoids (ACs), with TCs being more common.^5^, ^6^, ^7^, ^8^ Surgical resection is the preferred treatment approach for patients with localized PCs. Previous studies have reported that the long‐term survival rates in early PC patients were high after treated by local interventional therapy or surgery (lobar and sublobar resection, and pneumonectomy).^5^, ^9^ Our previous study also found that sublobar resection could achieve similar long‐term oncological outcomes with lobectomy in early‐stage PCs ≤3 cm in size when lymph node assessment was performed adequately.^10^ However, the physical condition and comorbidities of patients are also crucial determinants of overall survival (OS).^11^, ^12^ For those with acute or severe common illnesses, such as cardiovascular or cerebrovascular diseases, surgical intervention may not be the primary treatment option. Therefore, it is important to know the long‐term survival outcome for those PC patients without surgical resection. This study compared the outcomes of observation and surgical resection in patients with early‐stage PC tumors ≤3 cm in size without lymph node or distant metastasis using the data from the population‐based Surveillance, Epidemiology, and End Results (SEER) registry.

## 2 METHODS

### 2.1 Study population and data collection

The study was conducted in accordance with the Declaration of Helsinki (as revised in 2013). Data of all PC patients with a tumor diameter ≤ 3 cm without lymph node or distant metastasis were retrieved from the SEER database between 2000 and 2019, based on the International Classification of Diseases for Oncology, 3rd edition (ICD‐O‐3) using the National Cancer Institute SEER*Stat version 8.4.1 (http://www.SEER.cancer.gov/seerstat). Patients were excluded for any of the following criteria: (a) history of other malignancies; (b) diagnosed at autopsy or by death certificate only; (c) follow‐up status unavailable; and (d) history of treatment by radiotherapy or chemotherapy.

### 2.2 Statistical analyses

The primary outcomes consisted of OS and lung carcinoid‐specific survival (LCSS). OS was defined as the time from the diagnosis of PC to death due to any cause or the date of the last follow‐up. LCSS was calculated as the time from the diagnosis of PC to death due to PC. Deaths from causes other than PC were treated as censored observations. The baseline characteristics of the patients were summarized using descriptive statistics and compared using a chi‐squared test, where appropriate, for categorical variables and a *t*‐test for continuous variables. Kaplan–Meier curves and a log‐rank test were used to compare LCSS and OS between groups. To reduce the inherent bias of retrospective studies, propensity score matching (PSM) was performed at a 1:4 fixed ratio nearest‐neighbor matching between the observation and surgery groups for PC using the “MatchIt” package in R version 3.6.1 (cran.r‐project.org).^13^ Cox proportional hazard regression models were performed to determine factors associated with the risk of death in the overall population. The results are presented as the hazard ratio (HR) and 95% confidence interval (CI). Variables with *p* < 0.05 in the univariate analysis were included in the multivariate model. Two‐sided *p* < 0.05 was considered significant. Statistical analyses were performed using SPSS version 24 (SPSS Inc., Chicago, IL, USA) and plotted with GraphPad Prism 7.0 (GraphPad Software, La Jolla, CA, USA).

## 3 RESULTS

### 3.1 Patient characteristics

**FIGURE 1:**
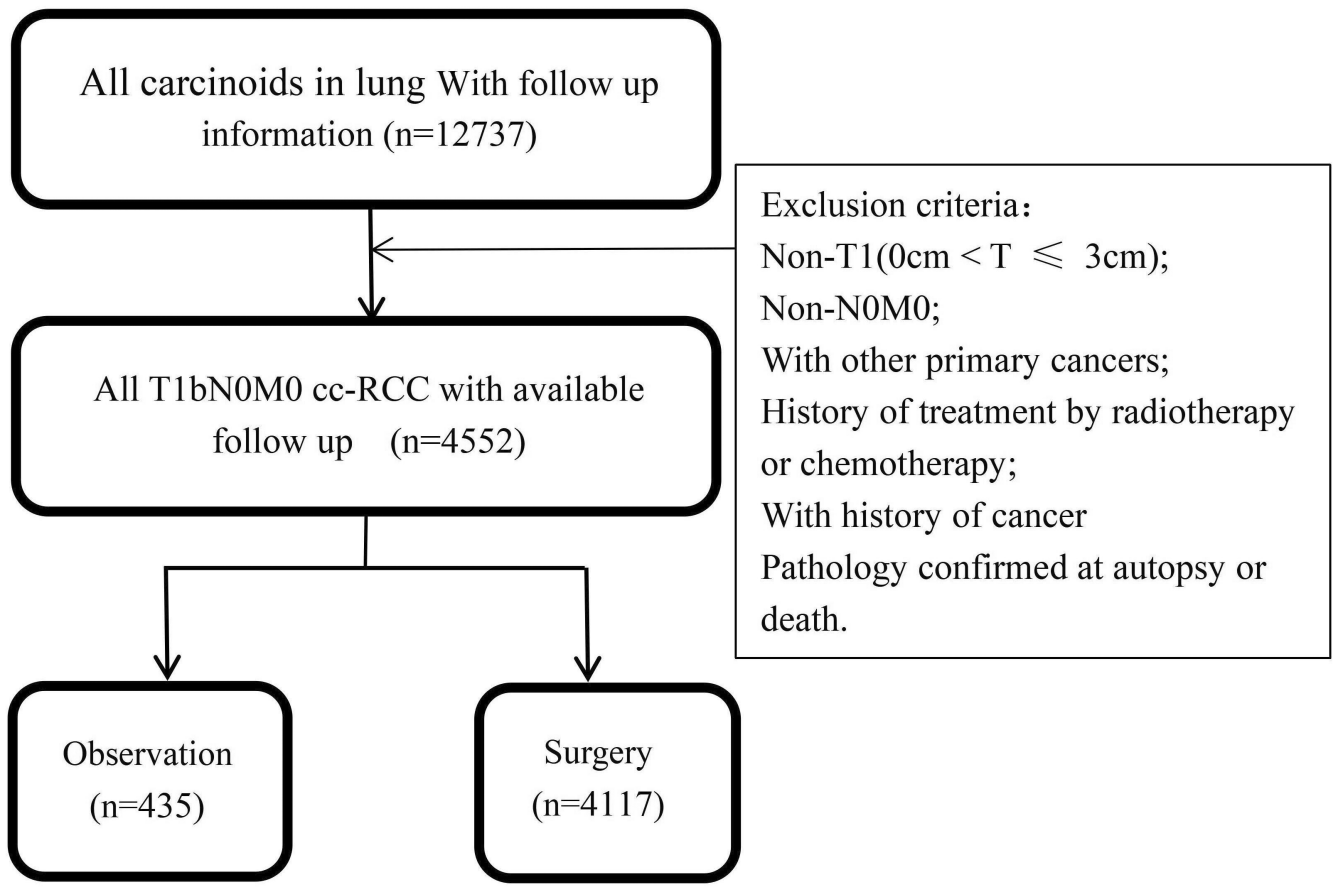
Flowchart for patient extraction from the Surveillance, Epidemiology, and End Results (SEER) database according to the inclusion and exclusion criteria.

We retrieved the data of 4552 PC patients using SEER software *Stat and the extraction process is shown in Figure 1 according to the inclusion and exclusion criteria. The clinicopathological characteristics of all enrolled PC patients are summarized in Table 1. In total, 4552 eligible PC patients, including 4122 (90.55%) with TCs and 430 (9.45%) with ACs, were enrolled. Among them, 4117 (90.44%) patients underwent surgical resection and 435 (9.56%) were observed (Table 1). Chi‐square testing revealed significantly more old patients in the observation group than in the surgery group.

**TABLE 1:** Clinicopathological characteristics of PCs before and after PSM.

Characteristics	All (*n* = 4552)	Observation (435)	Surgery (*n* = 4117)	*p*‐value	All (*n* = 1942)	Observation (432)	Surgery (*n* = 1510)	*p*‐value
Age								
≤65 years	2388 (52.46)	109 (25.06)	2279 (55.36)	<0.001	543 (27.96)	109 (25.23)	434 (28.74)	0.152
>65 years	2164 (47.54)	326 (74.94)	1838 (44.64)		1399 (72.04)	323 (74.77)	1076 (71.26)	
Sex								
Female	3297 (72.43)	327 (75.17)	2970 (72.14)	0.178	1418 (73.02)	325 (75.23)	1093 (72.38)	0.240
Male	1255 (27.57)	108 (24.83)	1147 (27.86)		524 (26.95)	107 (24.77)	417 (27.62)	
Race								
White	4104 (90.16)	385 (88.51)	3719 (90.33)	0.173	1742 (89.70)	382 (88.43)	1360 (90.07)	0.091
Black	291 (6.39)	38 (8.74)	253 (6.15)		126 (6.49)	38 (8.80)	88 (5.83)	
Other	131 (2.88)	11 (2.53)	120 (2.91)		61 (3.14)	11 (2.55)	50 (3.31)	
Unknown	26 (5.71)	1 (0.23)	25 (0.61)		13 (0.67)	1 (0.23)	12 (0.79)	
Histological type								
TC	4122 (90.55)	406 (93.33)	3716 (90.26)	0.037	1812 (93.31)	405 (93.75)	1407 (93.18)	0.675
AC	430 (9.45)	29 (6.67)	401 (9.74)		130 (6.69)	27 (6.25)	103 (6.82)	
Tumor size								
0–1 cm	865 (19.00)	62 (14.25)	803 (19.50)	<0.001	308 (15.86)	62 (14.35)	246 (16.29)	0.490
1–2 cm	2076 (45.61)	229 (52.64)	1847 (44.86)		1027 (52.88)	228 (52.78)	799 (52.91)	
2–3 cm	1002 (22.01)	116 (26.67)	886 (21.52)		472 (24.30)	115 (26.62)	357 (23.64)	
Unknown	609 (13.38)	28 (6.44)	581 (14.11)		135 (6.95)	27 (6.25)	108 (7.15)	
Grade								
Well differentiated	1484 (32.60)	78 (17.93)	1406 (34.15)	<0.001	383 (19.72)	77 (17.82)	306 (20.26)	0.394
Moderately differentiated	329 (7.23)	27 (6.21)	302 (7.34)		128 (6.59)	27 (6.25)	101 (6.69)	
Poorly differentiated	16 (0.35)	3 (0.69)	13 (0.32)		2 (0.10)	1 (0.23)	1 (0.07)	
Undifferentiated	5 (0.11)	0 (0)	5 (0.12)		0 (0)	0 (0)	0 (0)	
Unknown	2718 (59.71)	327 (75.17)	2391 (58.08)		1429 (73.58)	327 (75.69)	1102 (72.98)	
Tumor location								
Upper lobe	1442 (31.68)	143 (32.87)	1299 (31.55)	0.099	610 (31.41)	142 (32.87)	468 (30.99)	0.207
Middle lobe	984 (21.62)	79 (18.16)	905 (21.98)		389 (20.03)	78 (18.06)	311 (20.60)	
Lower lobe	1923 (42.25)	192 (44.14)	1731 (42.05)		838 (43.15)	191 (44.21)	647 (42.85)	
Other	119 (2.61)	8 (1.84)	111 (2.70)		61 (3.14)	8 (1.85)	53 (3.51)	
Unknown	84 (1.85)	13 (2.99)	71 (1.72)		44 (2.27)	13 (3.01)	31 (2.05)	
Laterality								
Right	2746 (60.33)	270 (62.07)	2476 (60.14)	0.254	1174 (60.45)	268 (62.04)	906 (60.00)	0.476
Left	1802 (39.59)	164 (37.70)	1638 (39.79)		765 (39.39)	163 (37.73)	602 (39.87)	
Unknown	4 (0.09)	1 (0.23)	3 (0.07)		3 (0.15)	1 (0.23)	2 (0.13)	
Treatment type								
Observation	435 (9.56)	435 (100)	0 (0)	<0.001	432 (22.25)	432 (100)	0 (0)	<0.001
Wedge	1027 (22.56)	0 (0)	1027 (24.95)		326 (16.79)	0 (0)	326 (21.59)	
Segmentectomy	261 (5.73)	0 (0)	261 (6.34)		97 (4.99)	0 (0)	97 (6.42)	
Sleeve	29 (0.64)	0 (0)	29 (0.70)		18 (0.93)	0 (0)	18 (1.19)	
Lobectomy	2684 (58.96)	0 (0)	2684 (65.19)		1017 (52.37)	0 (0)	1017 (67.35)	
Pneumonectomy	44 (0.97)	0 (0)	44 (1.07)		23 (1.18)	0 (0)	23 (15.23)	
Surgery, NOS	72 (1.58)	0 (0)	72 (1.75)		29 (1.49)	0 (0)	29 (1.92)	

Abbreviation: NOS, not otherwise specified.

Of the 4117 patients who underwent surgical resection, wedge resection was performed on 1027 (24.95%), segmentectomy on 261 (6.34%), sleeve resection on 29 (0.70%), lobectomy on 2684 (65.19%), and pneumonectomy on 44 (1.07%). To balance the two groups for subsequent analysis, the 432 patients in the observation group were propensity score matched 1:4 for age, histology type, tumor size, and grade to 1510 patients undergoing surgical resection. The baseline clinical and demographic characteristics for the matched cohorts are provided in Table 1. After PSM, there were no significant differences in each characteristic between the observation and surgery resection groups.

### 3.2 Outcome analysis before PSM

**FIGURE 2:**
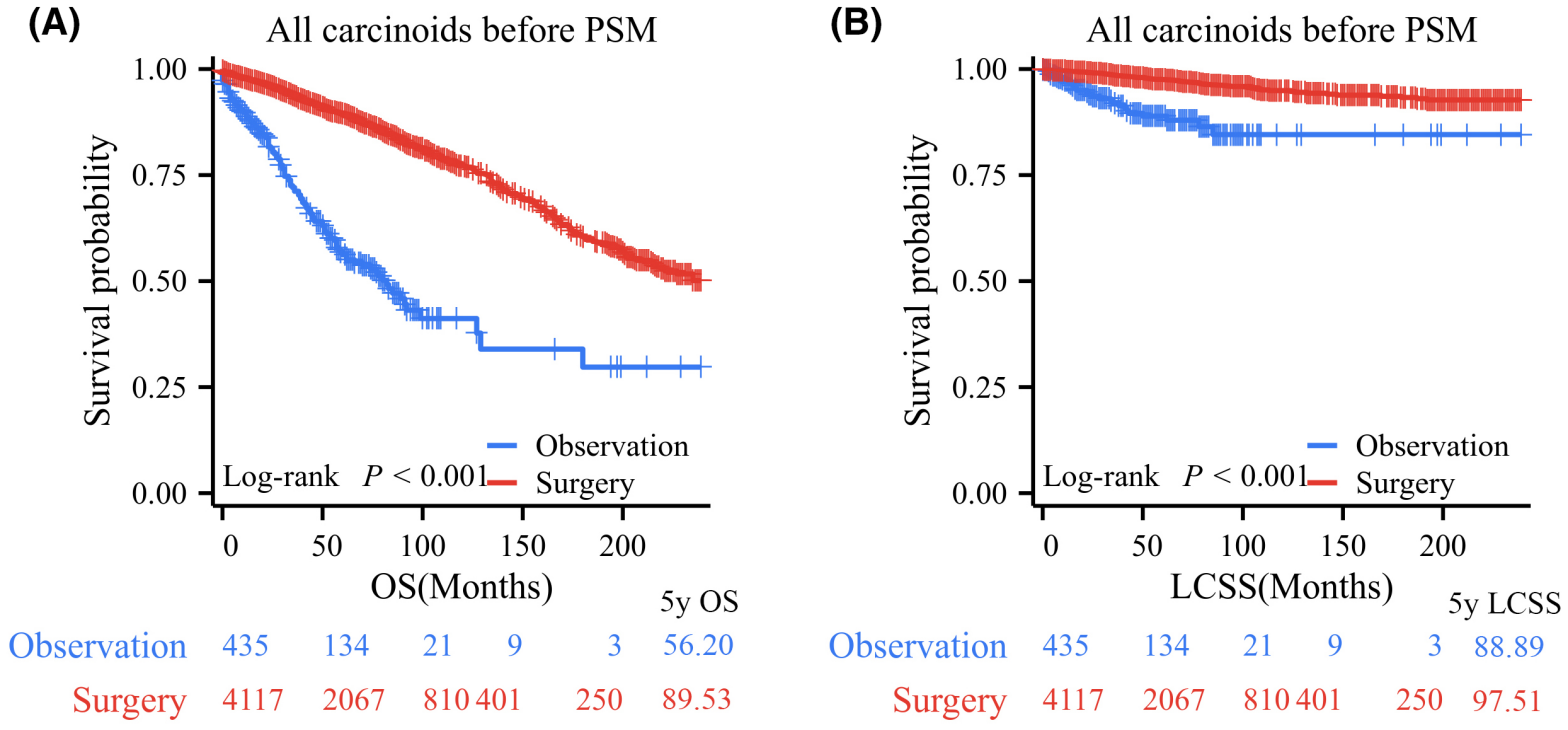
Kaplan–Meier survival curve of pulmonary carcinoids (PCs) before propensity score matching (PSM). (A) Surgical resection versus observation for (A) overall survival (OS) and (B) lung carcinoid‐specific survival (LCSS) in all PCs before PSM.

**FIGURE 3:**
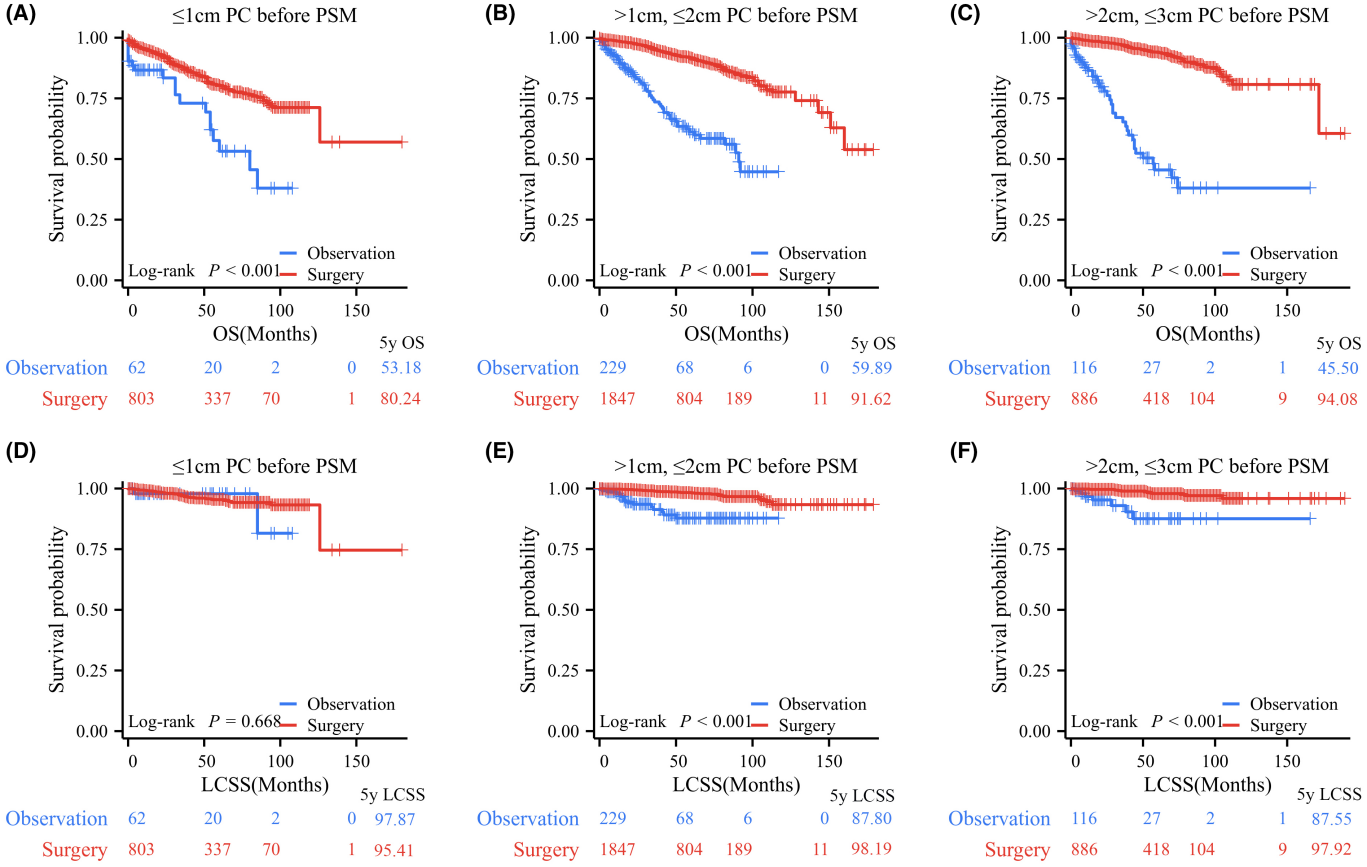
Kaplan–Meier survival curve of surgical resection versus observation in pulmonary carcinoids (PCs) stratified by tumor size before propensity score matching (PSM). (A) Surgical resection versus observation for overall survival (OS) in the T ≤ 1 cm PC group before PSM. (B) Surgical resection versus observation for OS in the *T* > 1 cm and ≤2 cm PC group before PSM. (C) Surgical resection versus observation for OS in the *T* > 2 cm and ≤3 cm PC group before PSM. (D) Surgical resection versus observation for lung carcinoid‐specific survival (LCSS) in the *T* ≤ 1 cm PC group before PSM. (E) Surgical resection versus observation for LCSS in the *T* > 1 cm and ≤2 cm PC group before PSM. (F) Surgical resection versus observation for LCSS in the *T* > 2 cm and ≤3 cm PC group before PSM.

Among all PC patients, log‐rank testing revealed that patients who underwent surgical resection had significantly better OS and LCSS than those who underwent observation, with 5‐year OS rates of 89.53% versus 56.20% and 5‐year LCSS rates of 97.51% versus 88.89% (Figure 2A). In terms of tumor size, before PSM, 865, 2076, and 1002 PC patients comprised the tumor (*T*) ≤ 1 cm, 1 cm < *T* ≤ 2 cm, and 2 cm < *T* ≤ 3 cm groups, respectively. Log‐rank test data indicated that patients in the surgery group had significantly longer OS than those in the observation group for PCs with *T* ≤ 1 cm (*p* < 0.001, 5‐year OS rates: 80.24% vs. 53.18%), 1 cm < *T* ≤ 2 cm (*p* < 0.001, 5‐year OS rates: 91.62% vs. 61.14%), and 2 cm < *T* ≤ 3 cm (*p* < 0.001, 5‐year OS rates: 94.08% vs. 45.50%; Figure 3A–C). Regarding LCSS, the log‐rank test revealed no significant difference between the surgery and observation groups for PCs with *T* ≤ 1 cm (*p* = 0.668, 5‐year LCSS rates: 97.87% vs. 95.41%; Figure 3D). However, PC patients who underwent surgery had a significantly better OS than those who underwent observation in the 1 cm < *T* ≤ 2 cm group (*p* < 0.001, 5‐year LCSS rates: 98.19% vs. 87.80%) and the 2 cm < *T* ≤ 3 cm group (*p* < 0.001, 5‐year LCSS rates: 97.92% vs. 87.55%; Figure 3E,F).

To further study the influence of surgery in PCs with different histology, we performed survival analyses in TCs and ACs separately and in subgroup analyses stratified by tumor size (Figures S1 and S2). For all TCs, the surgery group had significantly better LCSS and OS than the observation group (Figure S1). The subgroup analyses indicated that TC patients treated by surgery had longer OS than those who were observed for *T* ≤ 1 cm, 1 cm < *T* ≤ 2 cm, and 2 cm < *T* ≤ 3 cm, and 138, 168, and 68 patients died in the three subgroups, respectively. However, for the *T* ≤ 1 cm TC group, those who were observed displayed comparable LCSS with the surgery group (*p* = 0.626). The surgery group had longer LCSS than the observation group for 1 cm < *T* ≤ 2 cm and 2 cm < *T* ≤ 3 cm group TCs, and 27, 31, and 15 patients died of lung carcinoids in the three subgroups, respectively. For all ACs, the surgery group had significantly longer LCSS and OS than the observation group. Log‐rank tests indicated that the surgery group had longer OS than the observation group in the three size subgroups, and 13, 42, and 29 patients died in the three subgroups, respectively. Concerning LCSS, AC patients treated by surgery had better outcomes in the 1 cm < *T* ≤ 2 cm group, and for AC with *T* ≤ 1 cm and 2 cm < *T* ≤ 3 cm two groups had a comparable LCSS, and 4, 16, and 6 patients died of lung carcinoids in the three subgroups, respectively (Figure S2).

### 3.3 Outcome analysis after PSM

**FIGURE 4:**
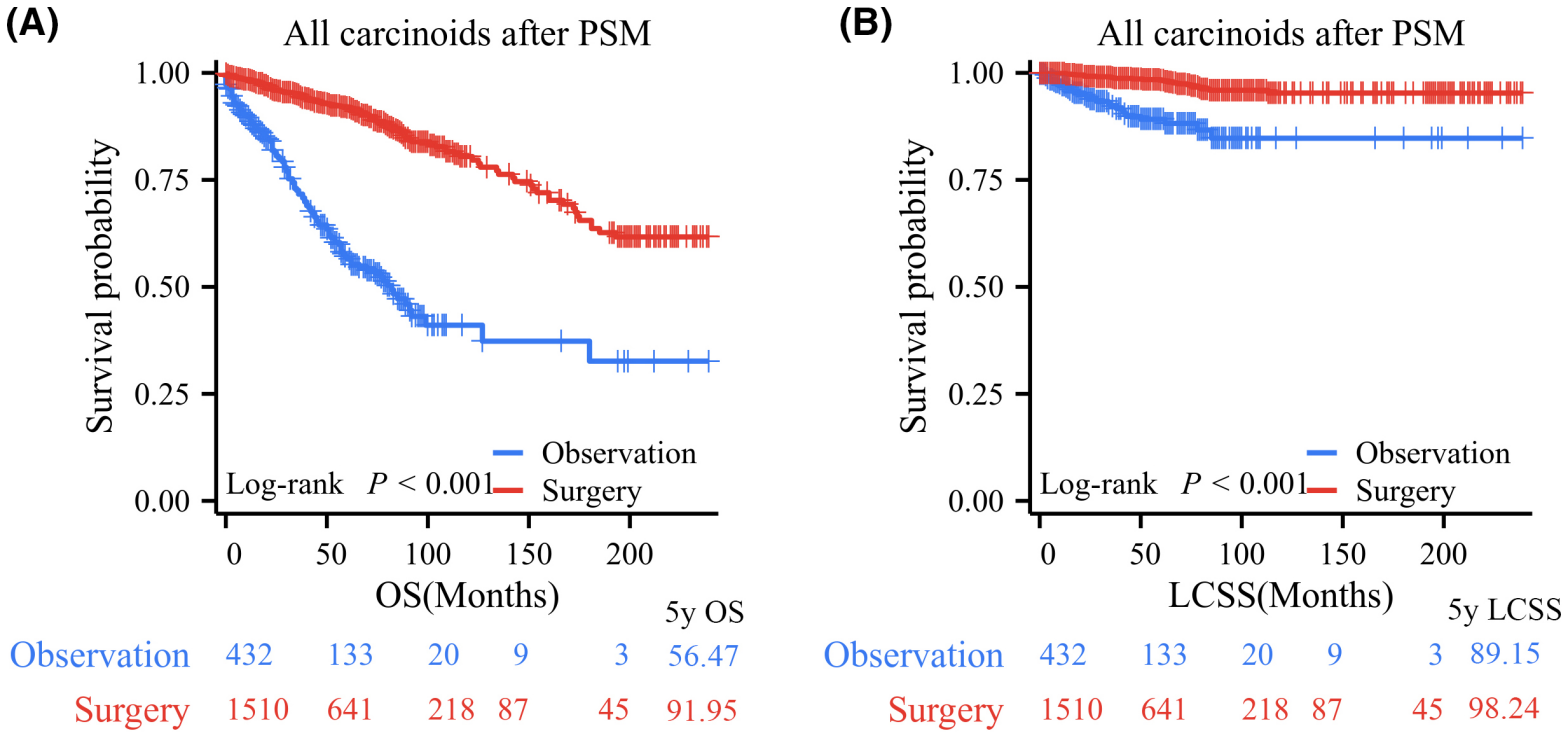
Kaplan–Meier survival curve of pulmonary carcinoids (PCs) after propensity score matching (PSM). Surgical resection versus observation for (A) overall survival (OS) and (B) lung carcinoid‐specific survival (LCSS) in all PCs after PSM.

**FIGURE 5:**
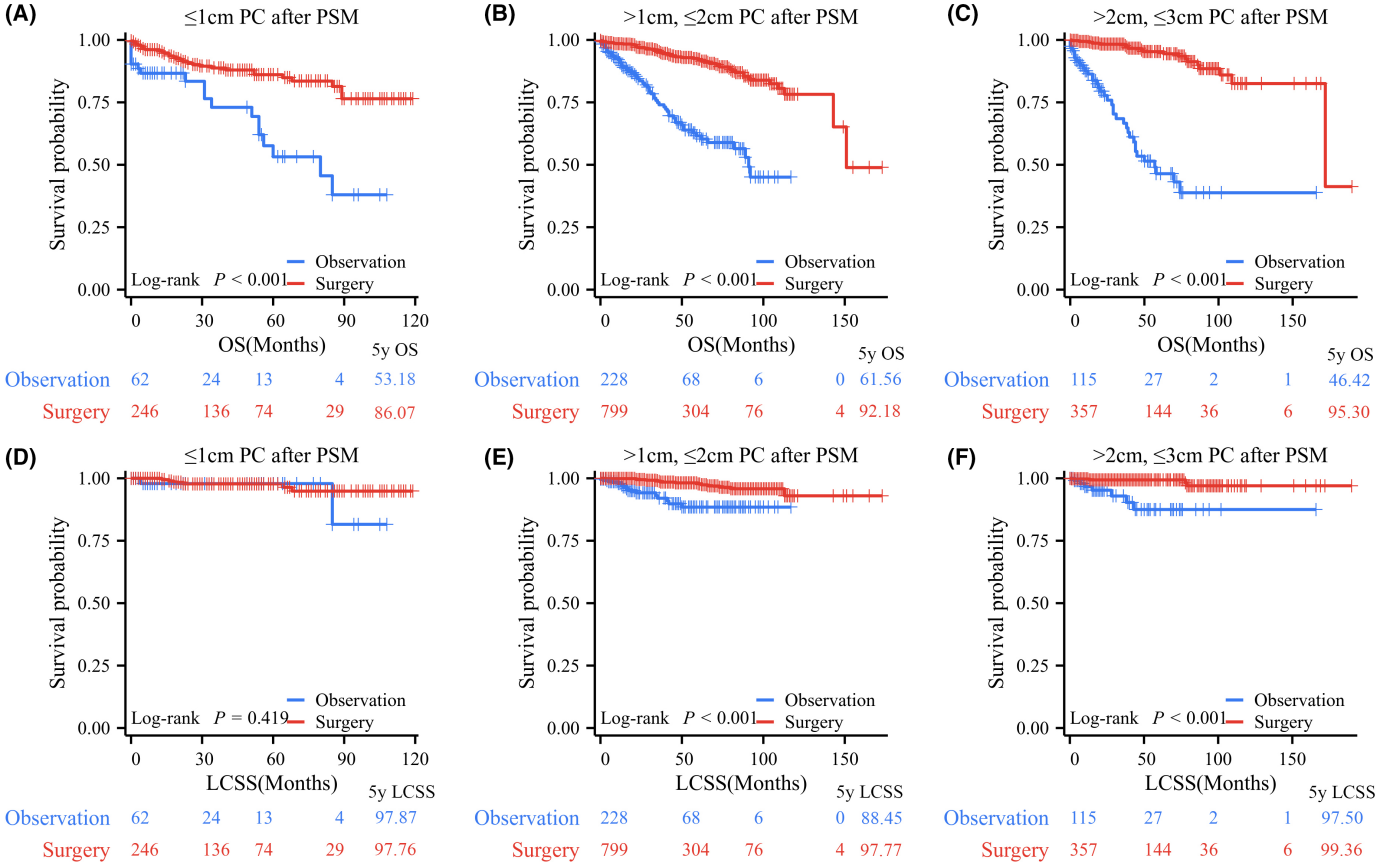
Kaplan–Meier survival curve of surgical resection versus observation in pulmonary carcinoids (PCs) stratified by tumor size after propensity score matching (PSM). (A) Surgical resection versus observation for overall survival (OS) in the *T* ≤ 1 cm PCs group after PSM. (B) Surgical resection versus observation for OS in the *T* > 1 cm and ≤2 cm PCs group after PSM. (C) Surgical resection versus observation for OS in the *T* > 2 cm and ≤3 cm PCs group after PSM. (D) Surgical resection versus observation for lung carcinoid‐specific survival (LCSS) in the *T* ≤ 1 cm PCs group after PSM. (E) Surgical resection versus observation for LCSS in the *T* > 1 cm and ≤2 cm PCs group after PSM. (F) Surgical resection versus observation for LCSS in the *T* > 2 cm and ≤3 cm PCs group after PSM.

After PSM, the observation and surgery resection groups did not exhibit any significant differences in any of the examined characteristics. Their clinical characteristics are presented in Table 1. All patients in the surgery group had significantly better OS and LCSS than those in the observation group, with 5‐year OS rates of 91.95% versus 56.47% and 5‐year LCSS rates of 98.24% versus 89.15% (Figure 4). Further subgroup analyses indicated that OS in the surgery group was significantly better than that in the observation group based on tumor size demonstrated that for PC patients with *T* ≤ 1 cm (*p* < 0.001, 5‐year OS rates: 86.07% vs. 53.18%), 1 cm < *T* ≤ 2 cm (*p* < 0.001, 5‐year OS rates: 92.18% vs. 61.56%), and 2 cm < *T* ≤ 3 cm (*p* < 0.001, 5‐year OS rates: 95.30% vs. 46.42%; Figure 5A–C). LCSS in PC patients who underwent surgery was significantly superior to that of those who underwent observation in the 1 cm < *T* ≤ 2 cm group (*p* < 0.001, 5‐year LCSS rates: 97.77% vs. 88.45%) and 2 cm < *T* ≤ 3 cm group (*p* < 0.001, 5‐year LCSS rates: 99.36% vs. 87.50%), while patients in the observation group had similar LCSS to those in the surgery group for PCs with *T* ≤ 1 cm (*p* = 0.419, 5‐year LCSS rates: 97.87% vs. 97.76%; Figure 5D–F).

Furthermore, survival analyses were separately performed in TC and AC patients (Figures S3 and S4). After PSM, 432 and 1515 patients remained in the observation and surgery groups, respectively. TC patients with surgery had longer LCSS and OS than those who underwent observation (Figure S3). Size subgroup analyses demonstrated that TC patients with surgery had longer OS than those who underwent observation in the *T* ≤ 1 cm, 1 cm < *T* ≤ 2 cm, and 2 cm < *T* ≤ 3 cm groups. The observation group displayed similar LCSS with the surgery patients (*p* = 0.432) in the *T* ≤ 1 cm TCs group and longer LCSS than surgery patients in the 1 cm < *T* ≤ 2 cm and 2 cm < *T* ≤ 3 cm TC group. For all ACs, the surgery group had significantly longer LCSS and OS than the observation group. After PSM, only 27 AC patients remained in the observation group. Survival analyses data are presented in Figure S4.

### 3.4 Cox regression analysis

To further validate the impact for LCSS of surgical intervention and observation on patient subgroups with varying tumor sizes, we conducted Cox regression analyses for each size subgroup. For PCs with *T* ≤ 1 cm, univariate analyses revealed that advanced age and poor differentiation were significantly associated with worse LCSS outcomes. However, subsequent multivariate analyses demonstrated that only age remained independently associated with LCSS outcomes (*p* = 0.009; HR = 3.098; 95% CI, 1.332–7.204) (Table 2). In the 1 cm < *T* ≤ 2 cm group, multivariate analyses indicated that advanced age (*p* < 0.001; HR = 4.092; 95% CI, 1.992–8.403), AC (*p* < 0.001; HR = 5.845; 95% CI, 3.079–11.097), and observation rather than surgery (*p* < 0.001; HR = 5.567; 95% CI, 2.859–10.841) were associated with worse LCSS outcomes (Table S1). Multivariate analyses indicated that advanced age (*p* = 0.016; HR = 3.267; 95% CI, 1.249–8.546), AC (*p* = 0.029; HR = 2.956; 95% CI, 1.12–7.8), tumor located on the left side (*p* = 0.007; HR = 3.535; 95% CI, 1.407–8.886), and observation rather than surgery (*p* < 0.001; HR = 6.829; 95% CI, 2.598–17.95) were associated with shorter LCSS in the 2 cm < *T* ≤ 3 cm group (Table S2).

**TABLE 2:** Univariate and multivariate Cox regression analyses of factors affecting lung carcinoid specified‐survival (LCSS) in carcinoid patients with tumors ≤1 cm.

	Univariate				Multivariate			
	HR	LL	UL	*p*	HR	LL	UL	*p*
Age								
>65 years versus ≤65 years	3.098	1.332	7.204	0.009	3.098	1.332	7.204	0.009
Sex								
Female								
Male	1.364	0.625	2.98	0.436				
race								
White								
Black	0.855	0.204	3.592	0.831				
Location								
Upper lobe								
Middle lobe	0.698	0.271	1.799	0.457				
Lower lobe	0.509	0.223	1.164	0.11				
Histology								
TC								
AC	1.236	0.375	4.077	0.727				
Laterality								
Right								
Left	1.557	0.768	3.154	0.219				
Treatment								
Observation								
Surgical resection	1.368	0.325	5.749	0.669				

## 4 DISCUSSION

In this population‐based study of small PC tumors, surgical resection was generally associated with increased OS compared with observation. The finding supports surgical resection as the current gold standard of treatment for PC tumors. In contrast, for *T* ≤ 1 cm PC tumors without lymph node and distant metastases, patients in the observation group had a comparable LCSS compared to patients who underwent surgical intervention. The 5‐year LCSS of patients with *T* ≤ 1 cm PC tumors without lymph node and distant metastases who underwent observation was remarkably high at 97.87%. The current findings align with a previous study that reported an average doubling time of approximately 7 years for carcinoid tumors.

Concerning the clinical features of patients, patients who did not receive surgery treatment were significantly older, consistent with a previous study.^5^ Age and other unmeasured variables may have led to decreased survival rates in patients undergoing observation. The 10‐year OS for patients undergoing surgical intervention was 77.19%. The 10‐year OS for patients who were observed was significantly lower at 41.17%. However, the 10‐year LCSS for patients who received surgery or who were observed was 94.92% versus 84.55%, respectively. These findings suggest that a higher proportion of patients in the observation group succumbed to non‐carcinoid tumor‐related causes than those who underwent surgical intervention. Therefore, it is crucial to consider other causes of mortality for patients diagnosed with early‐stage carcinoid. Consistent with previous findings, we also observed a twofold higher prevalence of carcinoid tumors in women than in men; however, gender did not appear to impact prognosis.^5^, ^15^

Subgroup analyses based on tumor size revealed that the 5‐ and 10‐year LCSS for observed patients with *T* ≤ 1 cm carcinoid tumors were 97.87% and 81.56%, respectively. These findings are comparable to the results obtained from patients who underwent surgical interventions (5‐ and 10‐year LCSS: 95.41% and 93.23%, respectively). However, patients with 1 cm < *T* ≤ 2 cm and 2 cm < *T* ≤ 3 cm who underwent surgical intervention displayed exhibited significantly higher LCSS with statistic difference.

A previous study included 8435 patients with small PCs through the National Cancer Database and found that patients in the surgical resection group had significantly longer OS than those with observation, which is consistent with our study.^16^ Compared to their study, we included LCSS in addition to OS as part of the survival outcome and performed size‐subgroup analyses to explore the significance of observations in the treatment of early lung carcinoids and found that for patients with *T* ≤ 1 cm carcinoid tumors, the observation group demonstrated comparable LCSS to the surgery group.^16^

The present findings have significant implications for counseling patients with incidentally detected carcinoid tumors. With the widespread utilization of low‐dose computed tomography in physical examinations, an increasing number of small pulmonary nodules are being identified.^17^ Patients diagnosed with early carcinoid who can tolerate surgical resection have a significant survival benefit compared with no treatment. Patients with limited life expectancy or those at high risk for surgery may have excellent lung carcinoid‐specific long‐term survival without surgical resection. Especially for patients with *T* ≤ 1 cm carcinoids, those who were observed displayed a similar LCSS to those that underwent surgical intervention in the present study.

Multivariate Cox regression analyses data reconfirmed that observation had similar LCSS with surgical intervention only in patients with *T* ≤ 1 cm carcinoid tumors. This study also identified laterality of tumor location as a significant independent prognostic factor for carcinoid patients with 2 cm < *T* ≤ 3 cm. Also, patients with tumors located on the left side of the body displayed significantly worse LCSS than patients with right side tumors. The present findings are consistent with a real‐world study in Europe.^18^ However, the underlying mechanisms remain unknown, which needs further exploration.

As a retrospective study, several intrinsic limitations should be considered. Firstly, as limited by database, we did not have sufficient data on comorbidities to analyze the effect of this variable on survival in this study cohort. Secondly, inevitable bias is inherent in retrospective studies, although adjustments using PSM were performed. Thirdly, the SEER database does not provide the reasons why these patients with observation did not undergo surgery. Lastly, the SEER data do not provide any insights into the progression of symptoms over time or the impact on patients' quality of life if they choose not to undergo resection. These factors are crucial in influencing the comparison of survival between the two groups and should be factored into the decision‐making regarding continued expectant management.

In conclusion, this study represents that the prognosis of untreated PC tumors ≤3 cm in size is not particularly unsatisfactory, although surgical resection is associated with an improved survival. Especially, for patients with *T* ≤ 1 cm carcinoid tumors, the observation group demonstrated comparable LCSS to the surgery group. Surgical resection is still the primary treatment for early stage PCs; however, active observation may also have its merits for patients who are not suitable candidates for surgery, including with limited life expectancy and severe symptomatic illness.

## AUTHOR CONTRIBUTIONS

**Xiongfei Li:** Conceptualization (equal); data curation (equal); formal analysis (equal); investigation (equal); methodology (equal); software (equal); validation (equal); visualization (equal); writing – original draft (equal). **Fanfan Fan:** Data curation (equal); formal analysis (equal); investigation (equal); methodology (equal); software (equal); validation (equal); visualization (equal); writing – original draft (equal). **Xuewang Jia:** Data curation (equal); formal analysis (equal); investigation (equal); methodology (equal); software (equal); validation (equal); visualization (equal); writing – original draft (equal). **Lingqi Yang:** Data curation (equal); formal analysis (equal); methodology (equal); validation (equal); visualization (equal). **Jinling He:** Data curation (equal); investigation (equal); methodology (equal); validation (equal). **Weibo Cao:** Data curation (equal); formal analysis (equal); investigation (equal); methodology (equal). **Quanying Tang:** Data curation (equal); investigation (equal); methodology (equal); software (equal); visualization (equal). **Ji Che:** Investigation (equal); methodology (equal); validation (equal); visualization (equal). **Song Xu:** Conceptualization (supporting); data curation (equal); formal analysis (equal); funding acquisition (equal); investigation (equal); project administration (equal); resources (equal); supervision (equal); validation (equal); writing – review and editing (supporting).

## FUNDING INFORMATION

This study was supported by the National Natural Science Foundation of China (82172776), Tianjin Key Medical Discipline (Specialty) Construction Project (TJYXZDXK‐061B, TJWJ2022XK005), and Diversified Input Project of Tianjin National Natural Science Foundation (21JCYBJC01770).

## CONFLICT OF INTEREST STATEMENT

The authors declare that they have no conflict of interest.

## ETHICS STATEMENT

This retrospective chart review study involving human participants was in accordance with the ethical standards of the institutional and national research committee and with the 1964 Helsinki Declaration and its later amendments or comparable ethical standards.

## Supporting information


Figure S1.

Figure S2.

Figure S3:

Figure S4.

Table S1.

Table S2.

Data S1.

## Data Availability

The population‐based research was retrospectively operated with data from the SEER database, which incorporates national information on tumor samples from 17 large‐scale cancer registries and is open to public for cancer studies (https://seer.cancer.gov/).
