# Supplementary figures and images for: Prognosis of unresected versus resected early‐stage pulmonary carcinoid tumors ≤3 cm in size: A population‐based study
Source: Cancer Med. 2024 Jun 10;13(11):e7311. doi: 10.1002/cam4.7311 (PMC11163264; doi:10.1002/cam4.7311)

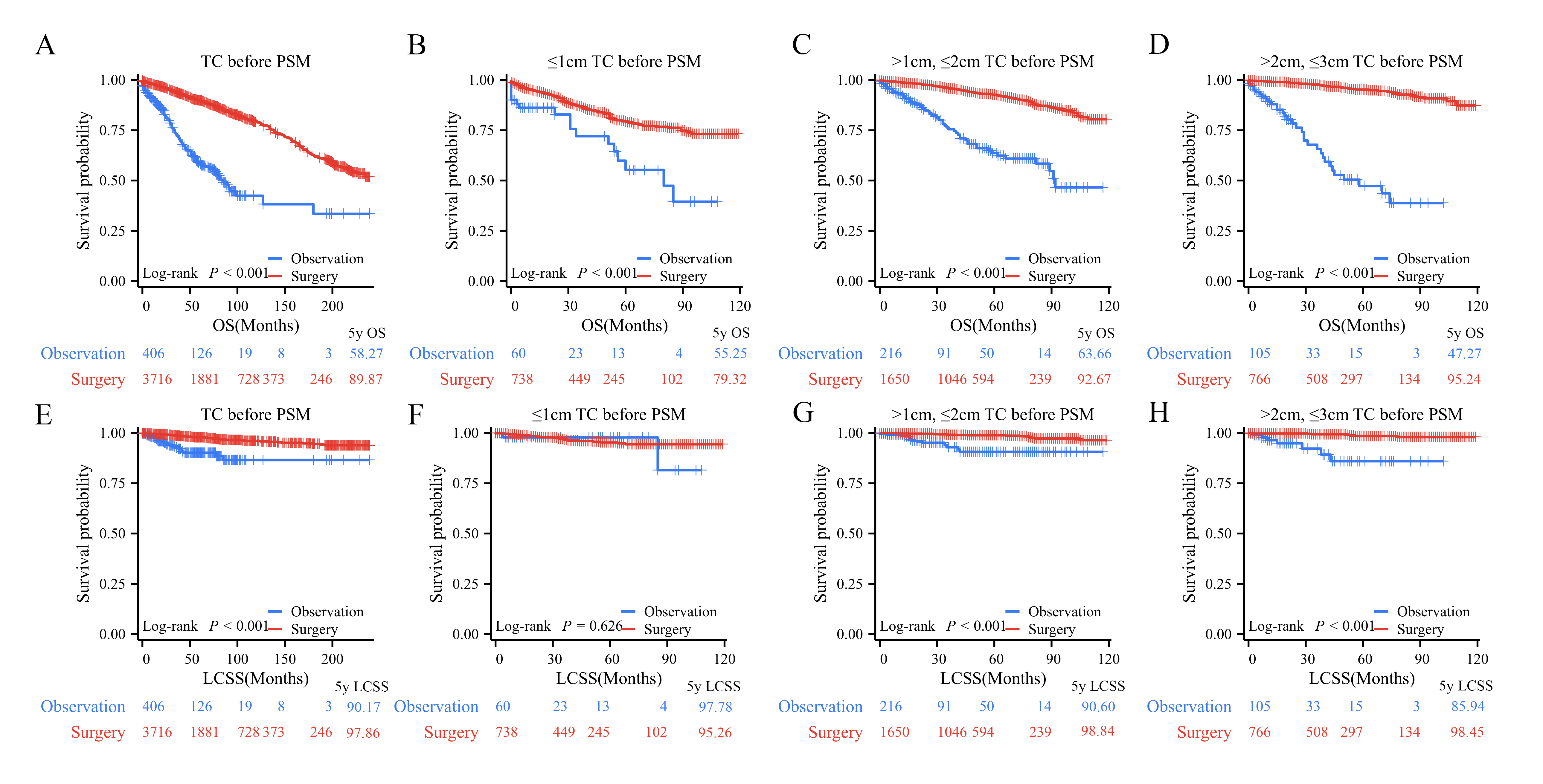

Supplement: Supplementary file 1 — Figure S1. [file CAM4-13-e7311-s007.jpg]

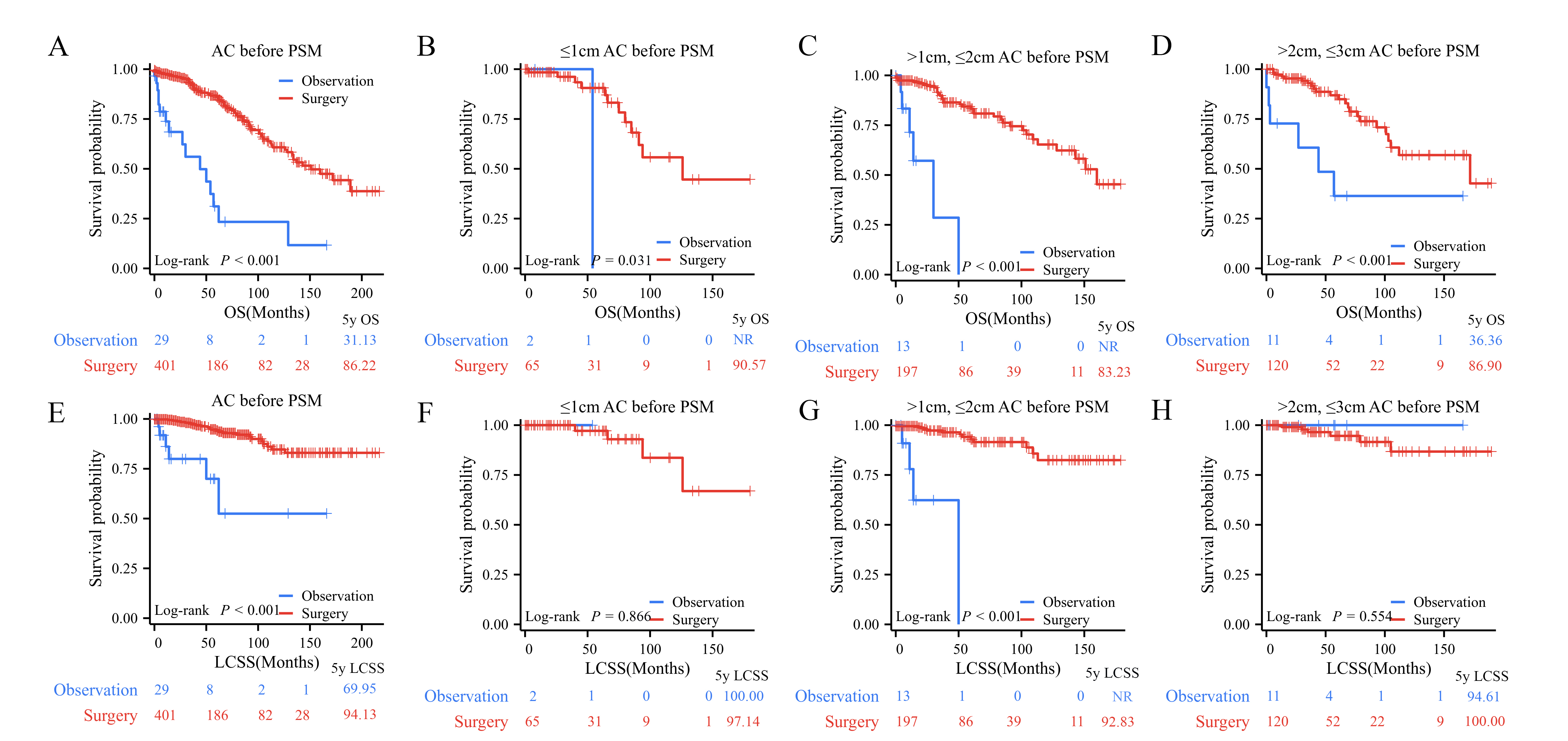

Supplement: Supplementary file 2 — Figure S2. [file CAM4-13-e7311-s002.jpg]

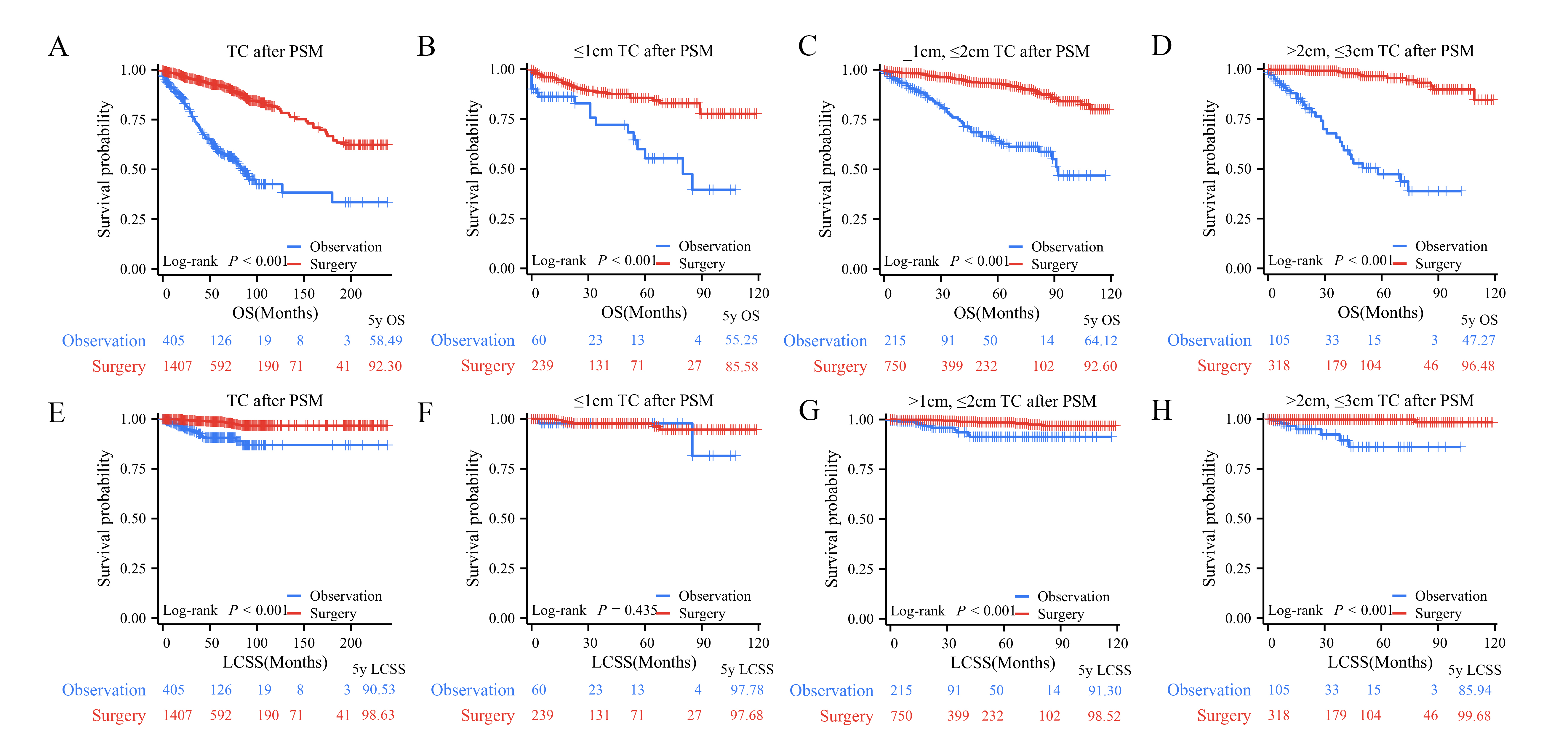

Supplement: Supplementary file 3 — Figure S3: [file CAM4-13-e7311-s004.jpg]

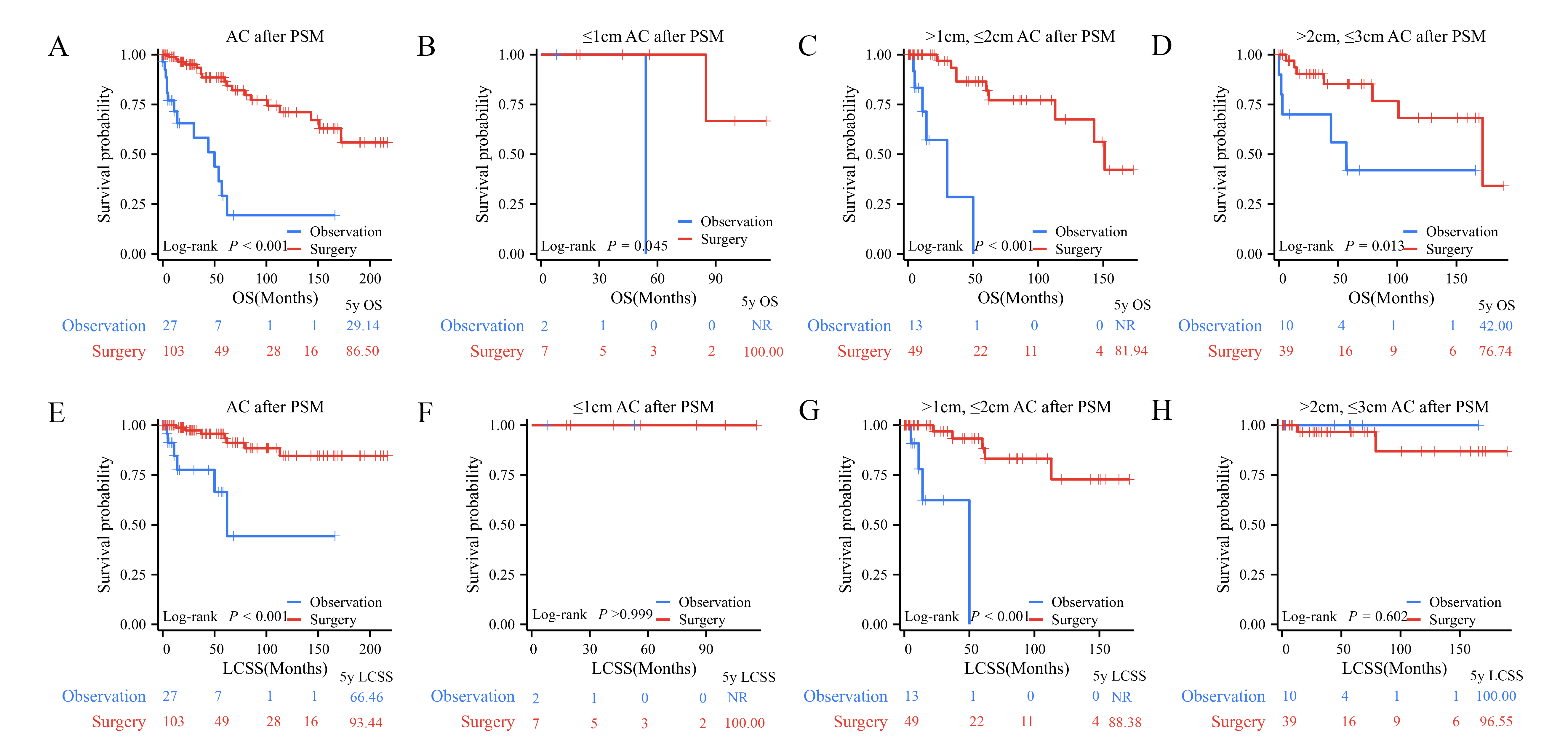

Supplement: Supplementary file 4 — Figure S4. [file CAM4-13-e7311-s005.jpg]
