# Supplementary material for: Prognosis of unresected versus resected early‐stage pulmonary carcinoid tumors ≤3 cm in size: A population‐based study
Source: Cancer Med. 2024 Jun 10;13(11):e7311. doi: 10.1002/cam4.7311 (PMC11163264; doi:10.1002/cam4.7311)
Supplement: Supplementary file 5 — Table S1. [file CAM4-13-e7311-s001.docx]

**e-Table 1:Univariate and multivariate Cox regression analyses of factors affecting**

**LCSS in carcinoid patients with tumors > 1 cm and ≤ 2 cm**

|  | Univariate | | | |  | Multivariate | | | |
| --- | --- | --- | --- | --- | --- | --- | --- | --- | --- |
|  | HR | LL | UL | P |  | HR | LL | UL | P |
| **Age** (>65y vs. ≤65y) | 5.012 | 2.485 | 10.108 | <0.001 |  | 4.092 | 1.992 | 8.403 | <0.001 |
| **Sex** (Female) |  |  |  |  |  |  |  |  |  |
| Male | 1.275 | 0.69 | 2.356 | 0.438 |  |  |  |  |  |
| **Race** (White) |  |  |  |  |  |  |  |  |  |
| Black | 0.797 | 0.193 | 3.294 | 0.754 |  |  |  |  |  |
| Other | 2.266 | 0.702 | 7.313 | 0.171 |  |  |  |  |  |
| **Location** (Upper lobe) |  |  |  |  |  |  |  |  |  |
| Middle lobe | 0.759 | 0.353 | 1.633 | 0.48 |  |  |  |  |  |
| Lower lobe | 0.703 | 0.362 | 1.368 | 0.3 |  |  |  |  |  |
| **Histology**(TC) |  |  |  |  |  |  |  |  |  |
| AC | 4.17 | 2.263 | 7.684 | <0.001 |  | 5.845 | 3.079 | 11.097 | <0.001 |
| **Laterality** (Right) |  |  |  |  |  |  |  |  |  |
| Left | 1.113 | 0.624 | 1.985 | 0.716 |  |  |  |  |  |
| **Treatment**(Surgery) |  |  |  |  |  |  |  |  |  |
| Observation | 5.766 | 3.103 | 10.715 | <0.001 |  | 5.567 | 2.859 | 10.841 | <0.001 |
