# Supplementary material for: Prognosis of unresected versus resected early‐stage pulmonary carcinoid tumors ≤3 cm in size: A population‐based study
Source: Cancer Med. 2024 Jun 10;13(11):e7311. doi: 10.1002/cam4.7311 (PMC11163264; doi:10.1002/cam4.7311)
Supplement: Supplementary file 6 — Table S2. [file CAM4-13-e7311-s006.docx]

**e-Table 2:Univariate and multivariate Cox regression analyses of factors affecting**

**LCSS in carcinoid patients with tumors > 2 cm and ≤ 3 cm**

|  | Univariate | | | |  | Multivariate | | | |
| --- | --- | --- | --- | --- | --- | --- | --- | --- | --- |
|  | HR | LL | UL | P |  | HR | LL | UL | P |
| **Age** (>65y vs. ≤65y) | 3.932 | 1.524 | 10.144 | 0.005 |  | 3.267 | 1.249 | 8.546 | 0.016 |
| **Sex** (Female) |  |  |  |  |  |  |  |  |  |
| Male | 1.814 | 0.77 | 4.271 | 0.173 |  |  |  |  |  |
| **Race** (White) |  |  |  |  |  |  |  |  |  |
| Black | 0.756 | 0.101 | 5.656 | 0.786 |  |  |  |  |  |
| Other | 1.992 | 0.266 | 14.907 | 0.502 |  |  |  |  |  |
| **Location** (Upper lobe) |  |  |  |  |  |  |  |  |  |
| Middle lobe | 0.318 | 0.07 | 1.452 | 0.139 |  |  |  |  |  |
| Lower lobe | 0.601 | 0.244 | 1.48 | 0.268 |  |  |  |  |  |
| **Histology**(TC) |  |  |  |  |  |  |  |  |  |
| AC | 2.497 | 0.966 | 6.456 | 0.059 |  | 2.956 | 1.12 | 7.8 | 0.029 |
| **Laterality** (Right) |  |  |  |  |  |  |  |  |  |
| Left | 3.077 | 1.242 | 7.627 | 0.015 |  | 3.535 | 1.407 | 8.886 | 0.007 |
| **Treatment**(Observation) |  |  |  |  |  |  |  |  |  |
| Surgical resection | 7.243 | 2.859 | 18.348 | <0.001 |  | 6.829 | 2.598 | 17.95 | <0.001 |
