# Supplementary material for: Prognosis of unresected versus resected early‐stage pulmonary carcinoid tumors ≤3 cm in size: A population‐based study
Source: Cancer Med. 2024 Jun 10;13(11):e7311. doi: 10.1002/cam4.7311 (PMC11163264; doi:10.1002/cam4.7311)
Supplement: Supplementary file 7 — Data S1. [file CAM4-13-e7311-s003.docx]

**e-**Figure 1**: Kaplan–Meier survival curve of surgical resection vs. observation in TCs stratified by tumor size before PSM.** A: Surgical resection vs. observation for OS in all TCs before PSM. B: Surgical resection vs. observation for OS in the T ≤ 1 cm TCs group before PSM. C: Surgical resection vs. observation for OS in the T > 1 cm and ≤2 cm TCs group before PSM. D: Surgical resection vs. observation for OS in the T > 2 cm and ≤3 cm TCs group before PSM. E: Surgical resection vs. observation for LCSS in all TCs before PSM. F: Surgical resection vs. observation for LCSS in the T ≤ 1 cm TCs group before PSM. G: Surgical resection vs. observation for LCSS in the T > 1 cm and ≤2 cm TCs group before PSM. H: Surgical resection vs. observation for LCSS in the T > 2 cm and ≤3 cm TCs group before PSM.

**e-**Figure 2**: Kaplan–Meier survival curve of surgical resection vs. observation in ACs stratified by tumor size before PSM.** A: Surgical resection vs. observation for OS in all ACs before PSM. B: Surgical resection vs. observation for OS in the T ≤ 1 cm ACs group before PSM. C: Surgical resection vs. observation for OS in the T > 1 cm and ≤2 cm ACs group before PSM. D: Surgical resection vs. observation for OS in the T > 2 cm and ≤3 cm ACs group before PSM. E: Surgical resection vs. observation for LCSS in all ACs before PSM. F: Surgical resection vs. observation for LCSS in the T ≤ 1 cm ACs group before PSM. G: Surgical resection vs. observation for LCSS in the T > 1 cm and ≤2 cm ACs group before PSM. H: Surgical resection vs. observation for LCSS in the T > 2 cm and ≤3 cm ACs group before PSM. NR:not reached, the follow-up time did not reach 5 years.

**e-**Figure 3**: Kaplan–Meier survival curve of surgical resection vs. observation in TCs stratified by tumor size after PSM.** A: Surgical resection vs. observation for OS in all TCs after PSM. B: Surgical resection vs. observation for OS in the T ≤ 1 cm TCs group after PSM. C: Surgical resection vs. observation for OS in the T > 1 cm and ≤2 cm TCs group after PSM. D: Surgical resection vs. observation for OS in the T > 2 cm and ≤3 cm TCs group after PSM. E: Surgical resection vs. observation for LCSS in all TCs after PSM. F: Surgical resection vs. observation for LCSS in the T ≤ 1 cm TCs group after PSM. G: Surgical resection vs. observation for LCSS in the T > 1 cm and ≤2 cm TCs group after PSM. H: Surgical resection vs. observation for LCSS in the T > 2 cm and ≤3 cm TCs group after PSM.

**e-**Figure 4**:Kaplan–Meier survival curve of surgical resection vs. observation in ACs stratified by tumor size after PSM.** A: Surgical resection vs. observation for OS in all ACs after PSM. B: Surgical resection vs. observation for OS in the T ≤ 1 cm ACs group after PSM. C: Surgical resection vs. observation for OS in the T > 1 cm and ≤2 cm ACs group after PSM. D: Surgical resection vs. observation for OS in the T > 2 cm and ≤3 cm ACs group after PSM. E: Surgical resection vs. observation for LCSS in all ACs after PSM. F: Surgical resection vs. observation for LCSS in the T ≤ 1 cm ACs group after PSM. G: Surgical resection vs. observation for LCSS in the T > 1 cm and ≤2 cm ACs group after PSM. H: Surgical resection vs. observation for LCSS in the T > 2 cm and ≤3 cm ACs group after PSM. NR:not reached, the follow-up time did not reach 5 years.
